# Public readiness for HIV self-testing in Kenya

**DOI:** 10.1080/09540121.2016.1191602

**Published:** 2016-06-02

**Authors:** Anna C. Heard, Annette N. Brown

**Affiliations:** ^a^ International Initiative for Impact Evaluation, 3ie, Washington, DC, USA

**Keywords:** HIV, self-test, Kenya, HIV testing and counseling

## Abstract

High interest and a growing body of evidence suggest that HIV self-testing could help fill the HIV testing gap for populations who have been hesitant to access testing services through current mechanisms. Evidence from five of six studies funded by 3ie answers questions posed by the Kenyan government to understand the readiness of Kenyans for HIV self-testing. The findings suggest that Kenyans are generally ready for HIV self-testing. Most people would not only like to obtain self-test kits through public health facilities but also expect to be able to obtain them from pharmacies – easy access being a key factor for a distribution outlet. Respondents across the studies seem to understand the importance of counseling and confirmatory testing, although the decision to access services after an HIV self-test will certainly be influenced by the results of the test. Respondents do have some concerns about potential harms and abuses from HIV self-tests. These concerns are focused on what they expect others would do, rather than reflections of what they say they would do themselves. Additionally, most people believe that such concerns were mostly unwarranted and/or could be addressed.

## Introduction

Calls for HIV self-testing to help fill the HIV testing gap have been increasing (Johnson et al., 2014; Myers, El-sadr, Zerbe, & Branson, 2013), and a growing body of evidence reveals high acceptance and perceptions of convenience and confidentiality among participants, including couples (Kumwenda et al., 2014) and healthcare workers (Kalibala et al., 2014). However, concerns of possible harms from HIV self-testing remain (Allais & Venter, 2014; Makusha et al., 2015; Scott, 2014).

In late 2012, the National AIDS and STI Control Programme (NASCOP) of the Ministry of Health agreed to collaborate with the International Initiative for Impact Evaluation (3ie) to promote the design and rigorous evaluation of projects using oral HIV self-tests. NASCOP wanted to learn whether HIV self-testing would help close the testing gap (2009), especially among those who had not previously tested. Citing concerns as above, NASCOP requested preliminary studies on the readiness of Kenyans for HIV self-testing. 3ie provided six teams grants to conduct research on questions raised by NASCOP, described below. In this article, we synthesize findings from five studies; complications with the data prevent us from including the sixth. The synthesis allows us to identify common patterns that may then be more representative of the entire population.

## Methods

We describe the five studies in Table 1. Individual study reports are available at http://www.3ieimpact.org. Four studies use large sample sizes (*N* = 239–2436), including both quantitative and qualitative data. One additionally samples men who have sex with men (MSM) and female sex workers (FSWs) separately. Three use random samples. One study uses a small sample, based on data saturation, with in-depth interviews and focus groups (Stankard, LeTouzé, & Jones, 2014). Only the accuracy study (Kurth et al., 2015) administered self-tests. No study attempts representative sampling for the entire Kenyan population.

**Table 1.** Features of included studies.

Authors	Primary institution	Study title	Sample size	Sample features	MSM and FSW	% male	% never tested	Survey dates
Kurth, Ann E and Siika, Abraham M	New York University	“Accuracy of oral HIV self-tests in Kenya: project description, findings, and recommendations”	239 (20 of which were video-taped)	Recruitment at clinics and at two workplaces in Eldoret (peri-urban)	N	36	10	November 2013
Stankard, Petra; LeTouzé, Olivier and Jones, Meghann	Population Services International	“How should HIV self-tests be packaged in Kenya?”	46: pre-mock-up: 5 FSW, 5 MSM, 16 General. Post: 4 FSW, 4 MSM, 12 general	Key informant interviews from various stakeholder groups in Mombasa (urban) and Siaya (rural)	Y	50	Unknown	October 2013 and January 2014
Ochako, Rhoune; Vu, Lung and Peterson, Katia	Population Services International	“Insights into potential users and messaging for HIV oral self-tests in Kenya”	782 general population + 100 MSM + 100 FSW	Household survey plus MSM and FSW surveys at drop-in clinics in Mombasa (urban) and Siaya (rural) using random sample	Y	45	12% of general population	November – December 2013
Okal, Jerry; Obare, Francis; Tun, Waimar and Matheka, James	Population Council	“Possible channels for distribution of HIV oral self-test kits in Kenya”	1436 general population + 317 service providers	Kisumu, Uasin Gishu, Nyandarua, Kilifi, and Nairobi using random sample Key informants also interviewed	N	34	11	October 2013
Kabiru, Caroline W.; Sidze, Estelle M., Egondi, Thaddaeus; Osok, Damar and Izugbara, Chimaraoke O.	African Population Health and Research Center	“Understanding the perceived social harms and abuses of oral HIV self-testing in Kenya: key findings of a cross-sectional study”	1133 survey + 118 in focus groups	Machako (rural), Korogocho and Viwandani (urban informal, Jericho and Harambee (urban formal) using random sample and for survey	N	50	20	August – November 2013

Each study addressed one main question but answered other questions. We provide a narrative synthesis of the studies, emphasizing areas of overlap where more than one study asked similar questions, which allows us to identify common patterns perhaps more representative of the entire population. We do not conduct statistical synthesis of the data, although we present some new analysis of the raw data, which were submitted as part of the grant requirements.

## Results

We present the results according to the basic questions addressed by different subsets of the surveys and tied to the questions of NASCOP.

### Would people use HIV self-test kits, and why or why not?

All four large-n studies report near universal acceptability (89–96%) among the general population, with similar rates for men and women. Three report specifically on those who have never tested. Acceptability is slightly lower, but still high (Supplemental Table A). In addition, Kabiru, Sidze, Egondi, Osok, and Izugbara (2014) find that when those who have never tested are asked – before discussing HIV self-tests – if they want to be tested for HIV, only 65% say yes. However, 84% of those never tested would “purchase and use” a self-test if it were available! Interest from key populations at drop-in clinics is mixed. Ninety-eight of 100 FSW, but only 57 of 100 MSM are interested (Ochako, Vu, & Peterson, 2014). Other demographic differences exist, but acceptability is generally above 90% (Ochako et al., 2014; Okal, Obare, Tun, & Metheka, 2014).

All five studies asked why people would use the test or about benefits. Ease of use or convenience/saving time, and privacy or confidentiality are commonly chosen (Ochako et al., 2014; Okal et al., 2014; Kabiru et al., 2014; Kurth & Siika, 2014; Stankard et al., 2014), as well as being “painless” with “no pricking” (Kurth & Siika, 2014) and providing empowerment (Stankard et al., 2014). Okal et al. (2014) report on reasons for not wanting to self-test, but they are generally not exclusive to HIV self-testing, excepting “never seen kit before” (Table 2). Ochako et al. (2014) provided four options and respondents largely chose “afraid of finding out positive result while alone”, with “health workers more knowledgeable”, “afraid of misinterpreting results” and “other reasons” half or less likely than the top reason.

**Table 2.** Reasons why respondents would not use a self-test kit (of those that would not use a self-test) from Okal et al. (2014).

	Percent of women *N* = 969	Percent of men *N* = 467	Percent of never tested *N* = 163	Percent of total *N* = 1436
Number who would not use a self-test	*n* = 64	*n* = 30	*n* = 22	*n* = 94
Low perception of risk	17	23	32	19
Never seen	17	17	5	17
Fear knowing/don’t want to know	17	13	27	16
Question accuracy	6	17	9	10
Already know HIV+	5	10	–	6
Don’t know how	6	3	5	5
I can’t pretend/not ready/don’t want/fear and feel could die/could make someone suicidal	3	10	–	5
No care/treatment/support	3	7	0	4
Unsure of cost	5	3	–	4
Need to consult spouse	6	0	0	4
Don’t know where to get care	3	3	5	3
Already tested	5	0	–	3
Don’t know where to get counseling	0	7	5	2
Don’t know where to get kit	3	0	0	2
Prefer facility	3	0	–	2
Takes too long	2	0	–	1
No reason	13	7	18	11

Note: The hyphen indicates that information about this reason for this sub-sample was not available. Source: Okal et al. (2014) and authors’ calculations.

### Does the oral HIV self-test kit work in Kenya?

Kurth and Siika (2014) provided self-test kits in a clinic setting with slightly modified package instructions but without individual demonstrations or coaching (Supplemental Figure A). Accuracy is based on comparison to a staff-administered rapid blood test, a proportion verified (with 100% correlation) by enzyme-linked immunosorbent assay (ELISA).

Sensitivity and specificity in the study are 89.7% and 99.4%, respectively. This compares well with manufacturer studies (91.7% and 99.9% respectively) under unobserved conditions (Orasure, 2012).

Kurth and Siika (2014) report that 15% of the oral fluid self-tests were invalid, higher than is deemed acceptable (∼2%) in the United States (FDA, 2012). All are due to user error, but known by the user to be invalid. Men have somewhat higher odds of invalid results (OR 2.74, *p* = .03). Limited information is available on errors. Of 20 video-taped testing experiences, only one was invalid. Recorded errors (invalid and not) were common, suggesting that package instructions alone may not be sufficient (Supplemental Table B).

### Where do people want to get self-test kits?

Okal et al. (2014) focused on distribution channels. Most respondents choose public health facilities both as the “preferred place” (64% of women, 59% of men) and when allowed multiple options (Table 3). Site preference is largely based on proximity (72%), followed by cost (23%). Kurth and Siika (2014) also report “easily available” as the strongest reason.

**Table 3.** Percent of participants who cite each type of facility as either the place where participant would be “most comfortable” obtaining the HIV self-test kit, or another location if the kit is not available at the “most preferred” place.

	Female (%) *N* = 974	Male (%) *N* = 472	Ever tested (%) *N* = 1278	Never tested (%) *N* = 167	Total (%) *N* = 1446
Government health facility	78	75	79	68	77
Private health facility	22	16	20	19	20
Pharmacy	23	20	23	18	22
Local administration	13	15	14	9	14
Shop or supermarket	12	13	12	13	12
Mobile clinic	13	10	13	6	12
Social marketing event	8	13	9	13	9
Community health worker	10	9	10	6	9
Faith-based or non-governmental health facility	9	9	9	7	9
School, church, or mosque	6	7	7	8	7
Community-based distributor	4	4	4	3	4
Family, friend, neighbor, etc.	4	8	5	10	5
Voluntary counseling and testing center	5	2	5	1	4
Community-based organization or self-help group	1	1	1	1	1
Non-governmental organization	0	0	0	0	0
Traditional birth attendant	1	0	1	0	0

Source: authors’ analysis of Okal et al. (2014) data.

Stankard et al. find that potential users expect pharmacies would distribute HIV self-test kits, likening kits to items such as family planning products. They rarely mention public health facilities, although when mentioned, they cite higher standards in the public sector (2014).

Providers in Okal et al. (2014) are overwhelmingly willing to provide information about HIV self-testing (97%) and distribute kits (93%).

### Will people seek services after using self-test kits?

Ochako et al. (2014) report that 61% of their general population sample and 40% of MSM and 75% of FSW would go to a clinic for a confirmatory test. Okal et al. (2014) find that 83% of women and 79% of men in their sample would seek counseling services after using an HIV self-test.

Kabiru et al. (2014) report that 74% select seek counseling, confirm results, or seek medication as the “one action they would most likely do” after a positive self-test.

### What are the concerns?

Kabiru et al. (2014) focused on potential social harms and designed their survey to elicit responses. They find that 61% of respondents think that HIV self-testing is open to abuse, with lower rates in rural areas (43%). Of listed abuses, the most selected are “intentionally infecting others”, “testing a partner without their consent”, and “parents testing their children”. However, only one responded *he* would “intentionally infect others” as his “main action”. Some abuses (testing without consent, disclosing others’ status) were thought more preventable than others (intentionally infecting others, parents testing children). When presented with a list of potential disadvantages, the most selected were people “might commit suicide” (32%) and “be anxious or depressed” (24%). However, only 9% report that *they* would go into depression as their main action if positive, and only 2% state that they would commit suicide (Kabiru et al., 2014). Note that these hypothetical responses contrast sharply with the evidence to date about observed harms from self-tests, which reveals no cases of suicide (Brown, Djimeu, & Cameron, 2014).

After HIV self-testing, participants in Kurth & Siika (2014) gave only positive responses for post-test actions, for example “wait, then repeat test” and “get support from health workers”.

When Ochako et al. asked about concerns related to a positive result without professional support, nearly a quarter raised some concern, including suicide and intentionally infecting others. However, even those who were fearful believed that “self-harm or harm to others was unwarranted” because “HIV is no longer an automatic death sentence” (2014, p. 18).

### What do Kenyans recommend?

Asked how to prevent potential abuses, respondents selected “make non-consensual testing illegal” (32%, although already illegal in Kenya), “self-test kit be used by the person availed to” (24%), “avail only one kit per person” (20%), and “sensitization” (16%) (Kabiru et al., 2014). Other survey respondents suggest awareness campaigns and educating people about procedures (Kurth & Siika, 2014) and recommend spreading the messages through mass media, health workers, and community outreach workers (Ochako et al., 2014). For packaging and labeling, influencers and stakeholders interviewed emphasized the importance of including information about counseling and confirmatory testing (Stankard et al., 2014).

## Discussion

Except for the accuracy results in Kurth and Siika (2014), studies are formative research based on surveys of knowledge, attitudes, and potential practice. Results for intent to seek services and other results are likely subject to social desirability bias. The format of the survey instrument for Kabiru et al. (2014) likely caused priming.

The findings suggest that Kenyans are generally ready for HIV self-testing. The vast majority of survey respondents view HIV self-testing as acceptable or would use HIV self-test kits, consistent with other literature from sub-Saharan Africa (e.g., Choko et al., 2011; Ng et al., 2012). The lack of difference by gender and by testing status suggests that self-tests could be acceptable for people who do not seek standard testing. People would like to obtain self-test kits through public health facilities but also expect to be able to obtain them from pharmacies. Primarily they want easy access. Perceptions of confidentiality and privacy are likely more related to the test result than obtaining the test kit. Certainly, seeking counseling and confirmatory testing after an HIV self-test will be influenced by the result. Nonetheless, respondents across the studies seem to understand their importance.

While concerns about potential harms from HIV self-tests exist, they are focused on what respondents expect others would do, rather than reflecting what they say they would do themselves. This is consistent with Brown et al. (2014) who find little if any evidence of such harms occurring.

Kenya is moving forward with HIV self-testing. Results from pilot studies distributing and using self-tests are forthcoming. Mechanisms for identifying and reporting negative consequences have been put in place.

Supplemental_materials.doc
