# Supplementary material for: Public readiness for HIV self-testing in Kenya
Source: AIDS Care. 2016 Jun 2;28(12):1528–32. doi: 10.1080/09540121.2016.1191602 (PMC5062035; doi:10.1080/09540121.2016.1191602)

# Supplemental materials

Table A. HIV self-testing acceptability rates

| Study | Question wording | Percent of  women | Percent of men | Percent of never-tested | Total  percent |
| --- | --- | --- | --- | --- | --- |
| Kurth et al. | Think/feel that HIV self-testing will be acceptable | - | - |  | 95 |
| Ochako et al. | Would use HIV oral self-test if made available | - | - | 92 | 96 |
| Okal et al. | Would use HIV oral self-test kits | 94 | 94 | 88* | 94 |
| Kabiru et al. | Would get an oral HIV self-test and do the test on their own | 89 | 93 | 84 | 91 |
| Stankard et. al. | Would you buy this HIV self-test kit?  Would you use this HIV self-test kit?  Would you recommend this HIV self-test kit to other people? | Potential users: generally enthusiastic | | | |

*author’s calculation of Okal et al.’s data.

**Table B. Test results and errors for video-taped participants**

| **Case** | **Sex** | **Self-test** | **Staff blood** | **User Errors** |
| --- | --- | --- | --- | --- |
| 1 | M | N | N | - Difficulty putting bottle in stand (holder)/Did not put bottle in stand - Did not swab between teeth and gums |
| 2 | F | N | N | - Difficulty opening bottle - Difficulty putting bottle in stand (holder)/Did not put bottle in stand - Put swab on table - Tongue touched swab |
| 3 | F | N | N | - Difficulty opening packet - Difficulty opening bottle - Holds swab in mouth - Waited < 20 minutes to read results |
| 4 | M | N | N | - Difficulty opening bottle - Difficulty putting bottle in stand (holder)/Did not put bottle in stand - Placed stand in wrong position - Spilled solution - Waited < 20 minutes to read results |
| 5 | M | I | N | - Difficulty opening bottle - Collected sample with finger instead of using swab - Placed finger in buffer solution before collecting sample with finger - Placed swab in buffer solution before collecting sample - Spilled solution - Waited < 20 minutes to read results |
| 6 | M | N | P | - Difficulty opening bottle - Put swab on table |
| 7 | M | N | N | - Difficulty opening bottle - Waited < 20 minutes to read results |
| 8 | F | N | N | - Difficulty opening bottle - Difficulty putting bottle in stand (holder)/Did not put bottle in stand - Placed swab in stand - Placed swab in buffer solution before collecting sample - Illiteracy - Did not keep swab in bottle for the entire time - Waited < 20 minutes to read results |
| 9 | M | N | N | - Difficulty opening bottle - Put swab on table |
| 10 | M | N | N | - Placed stand in wrong position |
| 11 | F | N | N | - Difficulty opening bottle - Waited < 20 minutes to read results |
| 12 | F | N | N | - Difficulty opening packet - Difficulty opening bottle - Touched bottom of swab with fingers - Used finger to swab with desiccant - Collected sample with finger instead of using swab - Placed swab in buffer solution without collecting sample - Did not keep swab in bottle for the entire time - Added desiccant to solution - Poured solution into stand - Spits into bottle - Illiteracy |
| 13 | M | N | N | - Difficulty opening bottle - Put swab on table - Waited < 20 minutes to read results |
| 14 | F | N | N | - Put swab on table - Waited < 20 minutes to read results |
| 15 | M | N | N | - Placed swab in stand - Touched bottom of swab with fingers - Waited < 20 minutes to read results |
| 16 | F | N | N | - Difficulty opening bottle - Put swab on table - Added desiccant to solution - Used swab as stirrer - Did not keep swab in bottle for the entire time - Waited < 20 minutes to read results |
| 17 | F | N | N | - Waited < 20 minutes to read results |
| 18 | F | N | N | - Difficulty opening bottle - Waited < 20 minutes to read results |
| 19 | F | N | N | - Difficulty putting bottle in stand (holder)/Did not put bottle in stand |
| 20 | M | N | N | - Difficulty opening bottle - Waited < 20 minutes to read results |

**Figure A: Modified package instructions**


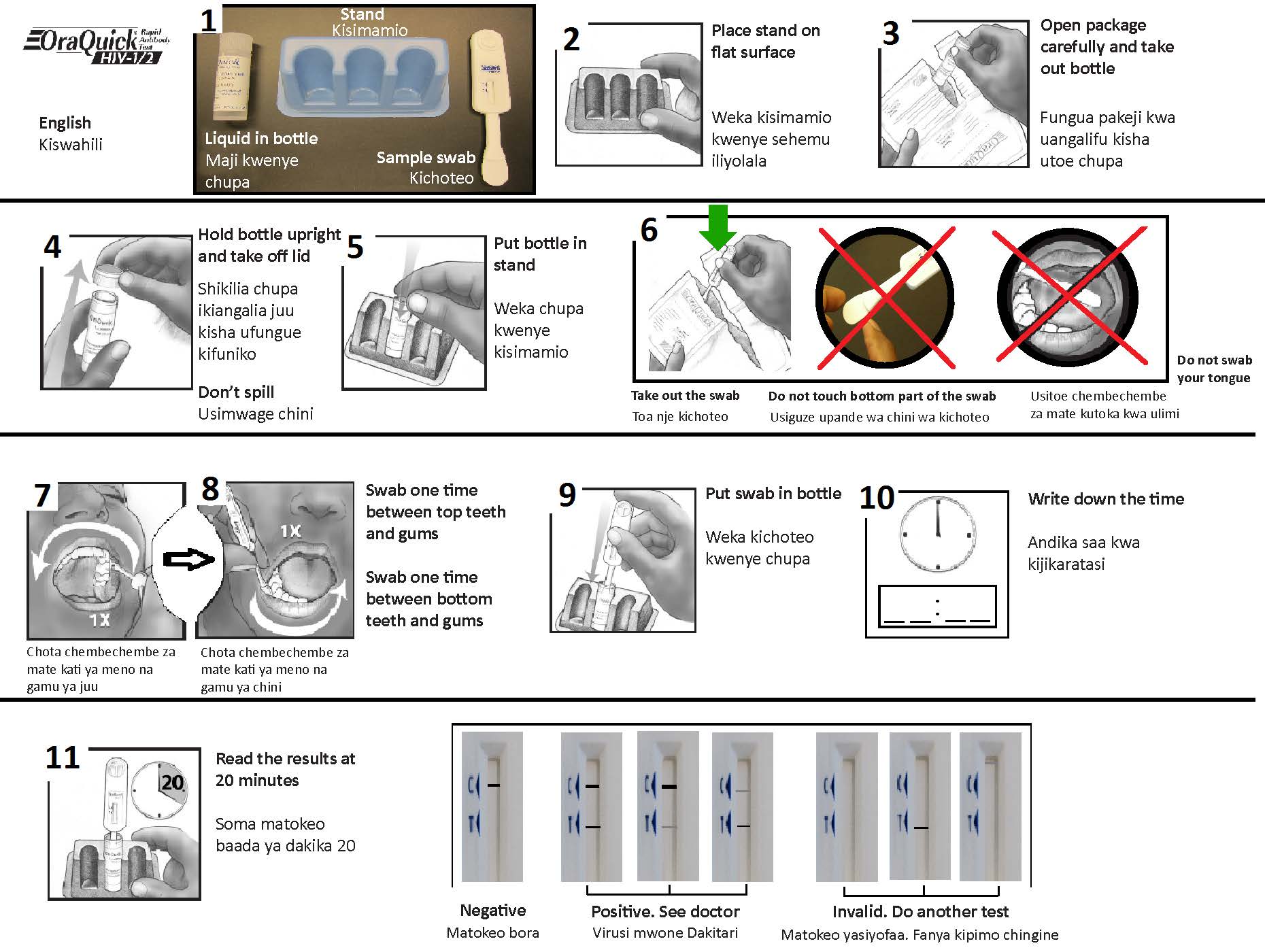

Supplement: Supplemental_materials.doc [file caic_a_1191602_sm0856.doc]
